# Regulation of Plant Growth and Development: A Review From a Chromatin Remodeling Perspective

**DOI:** 10.3389/fpls.2018.01232

**Published:** 2018-08-22

**Authors:** Simon P. Ojolo, Shijiang Cao, S. V. G. N. Priyadarshani, Weimin Li, Maokai Yan, Mohammad Aslam, Heming Zhao, Yuan Qin

**Affiliations:** ^1^Fujian Provincial Key Laboratory of Haixia Applied Plant Systems Biology, Key Laboratory of Genetics, Breeding and Multiple Utilization of Crops, Ministry of Education, State Key Laboratory of Ecological Pest Control for Fujian and Taiwan Crops, Fujian Agriculture and Forestry University, Fuzhou, China; ^2^College of Crop Science, Fujian Agriculture and Forestry University, Fuzhou, China; ^3^College of Forestry, Fujian Agriculture and Forestry University, Fuzhou, China; ^4^College of Resources and Environment, Fujian Agriculture and Forestry University, Fuzhou, China; ^5^College of Life Sciences, Fujian Agriculture and Forestry University, Fuzhou, China; ^6^College of Plant Protection, Fujian Agriculture and Forestry University, Fuzhou, China

**Keywords:** chromatin remodeling, SWI/SNF complexes, histones, gene regulation, meristem, hormone signaling, plant development, flowering

## Abstract

In eukaryotes, genetic material is packaged into a dynamic but stable nucleoprotein structure called chromatin. Post-translational modification of chromatin domains affects the expression of underlying genes and subsequently the identity of cells by conveying epigenetic information from mother to daughter cells. SWI/SNF chromatin remodelers are ATP-dependent complexes that modulate core histone protein polypeptides, incorporate variant histone species and modify nucleotides in DNA strands within the nucleosome. The present review discusses the SWI/SNF chromatin remodeler family, its classification and recent advancements. We also address the involvement of SWI/SNF remodelers in regulating vital plant growth and development processes such as meristem establishment and maintenance, cell differentiation, organ initiation, flower morphogenesis and flowering time regulation. Moreover, the role of chromatin remodelers in key phytohormone signaling pathways is also reviewed. The information provided in this review may prompt further debate and investigations aimed at understanding plant-specific epigenetic regulation mediated by chromatin remodeling under continuously varying plant growth conditions and global climate change.

## Introduction

The packaging of genetic material into nucleosomes is a distinctive evolutionary feature of eukaryotic cells. Nucleosomes are repetitive units consisting of 147 bp DNA strands tightly wrapped around a central octamer comprising one histone heterotetramer (H3/H4) and two histone heterodimers (H2A/H2B). Histones are highly conserved proteins found in all eukaryotes as core nucleosome units (Suzuki and Bird, 2008). Together, the histone octamer and DNA resemble “beads” on a string and are referred to as chromatin (Luger et al., 1997). This packaging ensures that long DNA strands are tightly condensed by supercoiling to precisely fit into the nucleus. Such compaction of nucleosomes in chromatin hinders DNA accessibility to important regulatory proteins essential for various nuclear processes. However, cells have evolved in response to this impediment through chromatin remodeling (Groth et al., 2007).

Gene regulatory mechanisms in eukaryotic cells (such as transcription, DNA repair and replication) act upon chromatin structure as a substrate. These activities induce cellular changes and regulate gene expression in a number of biological processes, including genome stability, recombination, developmental reprogramming and response to extracellular signals (Feng et al., 2010; Soria et al., 2012; Zhu et al., 2013). The regulatory precision of these fundamental genome processes relies on the high fidelity of chromatin remodeling mechanisms that permit temporal access to – or blocking of – vital DNA sequences, such as gene promoters. Histone modifications, nucleosome remodeling and DNA methylation have been shown to regulate chromatin structure and gene expression (Henikoff and Shilatifard, 2011).

There are two main players regulating chromatin dynamics: (1) chromatin remodelers that alter DNA-histone interactions by energy harnessed through ATP hydrolysis and (2) nucleosome-modifying enzymes that modulate DNA and histone residues by specifically adding or removing covalent modifications (Jerzmanowski, 2007). Organisms rely on gene expression regulation to achieve normal cell differentiation, organogenesis, growth and development. Moreover, gene expression regulation is temporally and spatially coordinated via crosstalk between chromatin remodeling complexes (CRCs) and the transcription machinery (Deem et al., 2012). In plants, such precise control of gene expression mediated by chromatin modifications in response to endogenous and environmental stimuli is fundamental for proper development and reproductive success (Sarnowski et al., 2005; Bezhani et al., 2007; Han et al., 2012; Archacki et al., 2013; Efroni et al., 2013; Sarnowska et al., 2013; Qin et al., 2014; Vercruyssen et al., 2014).

Plant development can be divided into the embryonic and postembryonic phases. The embryonic development phase includes the establishment of the seedling apical-basal axis, the shoot apical meristem (SAM) and the root apical meristem (RAM). The subsequent establishment of the leaf, stem and flower meristems occurs in the postembryonic phase. In this review, we discuss recent investigations underpinning the involvement of SWI/SNF chromatin remodeling ATPases in regulating key plant growth and development processes, including meristem establishment and maintenance, cell differentiation, organ initiation, flower morphogenesis and flowering time regulation. We also discuss the role of chromatin remodelers in the plant response to key phytohormone signals. This overview may be useful for framing the current knowledge gaps, thereby stimulating further debate and research aimed at a comprehensive understanding of epigenetic regulatory mechanisms in plant development. This in turn may guide molecular-based plant improvement techniques for desirable agronomic traits.

## Classification of Eukaryotic Chromatin Remodeling Complexes

Chromatin remodeling complexes are evolutionarily conserved multi-unit protein complexes that regulate chromatin structure by altering nucleosome composition and interactions (Narlikar et al., 2002). A common feature of all purified CRCs is that they harbor an ATPase/helicase of the SWITCHING DEFECTIVE2/SUCROSE NON-FERMENTING2 (SWI2/SNF2) family as their catalytic core. The SWI/SNF family is part of superfamily2 (SF2), a large family of helicases and translocases, and is named after the first identified CRC (Peterson et al., 1994). SWI/SNF CRCs utilize the energy derived from ATP hydrolysis to control accessibility to vital DNA sequences by influencing nucleosome structure and position and determining the kind of variant histone subspecies to be incorporated (Eisen et al., 1995; Clapier and Cairns, 2009).

A number of *Arabidopsis* SWI/SNF complex subunits have been identified based on sequence similarity with metazoan subunits. These include four SWI2/SNF2 ATPases (BRAHMA [BRM], SPLAYED [SYD], MINU1/CHR12 and MINU2/CHR23) (Knizewski et al., 2008; Han et al., 2015); four SWI3 proteins (SWI3A to SWI3D); two SWI/SNF ASSOCIATED PROTEINS 73 (SWP73A/CHC2 and SWP73B/CHC1, also called BRAHMA ASSOCIATED FACTOR 60 (BAF60) in humans); two ACTIN RELATED PROTEINS belonging to SWI/SNF complexes (ARP4 and ARP7); and the BUSHY (BSH) protein (Meagher et al., 2005; Jerzmanowski, 2007; Kwon and Wagner, 2007; Sang et al., 2012). Eukaryotic SWI2/SNF2 family chromatin remodelers can be categorized into four classes/subfamilies based on phylogenetic analysis, and all four of these subfamilies are represented in plants (Farrona et al., 2008).

### SWI/SNF Subfamily Remodelers

The SWI/SNF subfamily of remodelers initially purified from *Saccharomyces cerevisiae* is probably the most comprehensively studied. SWI/SNF subfamily CRCs have a well-established role in gene expression regulation. The SWI/SNF subfamily remodelers contain 8–14 subunits, and the catalytic ATPases of most SWI/SNF subfamily remodelers are composed of a helicase-SANT domain, a post-HSA domain and a C-terminal bromodomain. While the fungal SWI/SNF subfamily ATPases consist of a pair of actin-related proteins (ARPs) (Cairns et al., 1998), higher orthologs of SWI/SNF subfamily complexes contain a dimer of an ARP and actin (Lessard et al., 2007). The mode of action of SWI/SNF complexes has been characterized as sliding and/or ejecting nucleosomes at many target loci, but they reportedly have no vital role in the assembly of eukaryotic chromatin structure (Clapier and Cairns, 2009). It was recently reported that the *Arabidopsis* SWI/SNF complex expedites its role in activating and repressing target gene expression by binding to both promoters and terminators and that it regulates the expression of both promoter-centered genes and non-coding RNAs (Archacki et al., 2017).

### Imitation Switch (ISWI) Subfamily Remodelers

The ISWI subfamily remodelers have 2–4 subunits. Eukaryotic ISWI remodeler complexes typically have 1 or 2 distinct catalytic subunits and specialized attendant proteins that give rise to various domains. These domains include plant homeodomains, bromodomains, DNA-binding histone fold motifs and additional DNA-binding motifs (Clapier and Cairns, 2009). ISWI complexes were initially purified from *Drosophila melanogaster.* They include the nucleosome remodeling factor (dNURF), chromatin accessibility complex (dCHRAC) and ATP-utilizing chromatin assembly and remodeling factor (dACF) (Corona and Tamkun, 2004). SANT-like ISWI ATPases contain a SANT domain adjacent to a SLIDE domain in their C-terminus, which acts as a binding site for unmodified histone tails and DNA. The diversity resulting from attendant subunits is exhibited in the modes of action of the ISWI family complexes. Both ACF and CHRAC have been shown to promote chromatin assembly and transcription repression through the optimization of nucleosome spacing, whereas NURF assists RNA polymerase II (RNAPII) activation by randomizing nucleosome spacing (Corona and Tamkun, 2004).

### Chromodomain Helicase DNA-Binding (CHD) Subfamily Remodelers

The CHD subfamily remodelers were first purified from *Xenopus laevis* and comprise 1–10 subunits. The N-terminus of their catalytic subunit consists of two chromodomains arranged tandemly, with the composition varying from monomeric in lower eukaryotes to multimeric in vertebrates (Marfella and Imbalzano, 2007). CHD subfamily remodelers vary in their structure, composition and function, due in part to the diversity of their chromodomains. One of the members of the CHD subfamily in vertebrates, the nucleosome remodeling and deacetylase (Mi-2/NuRD) complex, has repressive roles due to its inclusion of histone deacetylases (HDAC1/2) and methyl CpG-binding domain (MBD) proteins. In contrast, other CHD subfamily remodelers have been reported to promote transcription by either ejecting or sliding nucleosomes along the DNA double strand (Denslow and Wade, 2007).

### Inositol Requiring 80 (INO80) Subfamily Remodelers

The INO80 subfamily is the most recently characterized chromatin remodeler subfamily. Many INO80 subunit homologs have been identified in yeast, fruit fly, mammals and plants, making it the most conserved subfamily. The presence of a split ATPase subunit distinguishes INO80 complexes from the SWI/SNF, CHD and ISWI subfamilies (Morrison and Shen, 2009). In *S. cerevisiae*, the INO80 subfamily is represented by two complexes, INO80 and Swi2/snf2-related 1 (SWR1); in mammals, it is represented by three complexes, INO80, Snf2-related CBP activator protein (SRCAP) and p400. *D. melanogaster* has INO80 and p400 complexes (Morrison and Shen, 2009; Bao and Shen, 2011). *S. cerevisiae* INO80 and SWR1 ATPases are approximately 1.2–1.5 MDa in mass and are reported to contain 15 and 14 subunits, respectively (Shen et al., 2000; Krogan et al., 2003; Mizuguchi et al., 2004). In general, the INO80 subfamily has a relatively conserved composition of individual complexes and a high degree of homology in the ATPase subunit. Studies of the *S. cerevisiae* SWR1 complex indicate the importance of a spacer (split) region in the ATPase subunit, as deletion of this region leads to dissociation of a number of subunits from the complex, including the RuvB-like Rvb1 and Rvb2 subunits (Wu et al., 2005). The INO80 subfamily is responsible for various functions in eukaryotic cells including transcriptional activation and DNA double-strand break (DSB) repair (Ebbert et al., 1999).

## Involvement of Swi/Snf Atpases in Meristem Establishment and Maintenance

Plant development is characterized by the presence of stem cells with the capacity to self-renew and transform into tissue-specific founder cells. The stem cells of the RAM and SAM have been widely studied in plants and are found at the root and shoot tips, respectively (Shen and Xu, 2009). Stem cell homeostasis and identity in both plants and mammals are associated with the activity of SWI/SNF chromatin remodeling ATPases (Ori et al., 2000; Kwon et al., 2005; Ho et al., 2009; Aichinger et al., 2011). Perturbations in the Polycomb group (PcG) repressive complex, histone acetylation or chromatin assembly may lead to improper RAM or SAM development (Phelps-Durr et al., 2005; Schubert et al., 2006; Barrero et al., 2007; Xu and Shen, 2008; Kornet and Scheres, 2009). Individual *Arabidopsis* SWI/SNF subfamily chromatin remodelers (BRM, SYD, CHR12 and CHR23) (Flaus et al., 2006) are also involved in regulating numerous plant developmental processes (Farrona et al., 2004; Hurtado et al., 2006; Kwon et al., 2006; Bezhani et al., 2007; Tang et al., 2008; Wu et al., 2012).

**FIGURE 1:**
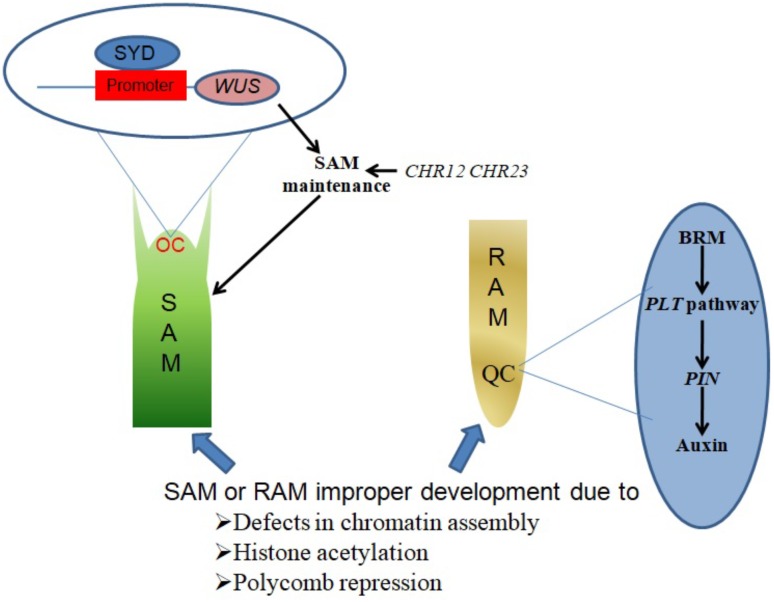
Chromatin remodelers in shoot and root apical meristem establishment and maintenance. SYD binds to the promoter region of *WUS* in the OC region of the SAM, activating *WUS* expression to promote SAM maintenance. CHR12 and CHR23 have redundant roles in plant development regulation, as single mutations in either produce no obvious defects but strong double mutations lead to impaired SAM maintenance. BRM acts in the *PLT* pathway to regulate the expression of *PIN* genes, which in turn influence auxin distribution in the QC region to ensure RAM maintenance.

SYD promotes SAM maintenance by binding to the promoter region of its target gene *WUSCHEL* (*WUS*) and activating *WUS* expression in the organizing center (OC) region of the SAM (**Figure 1**). Consistent with this function, *Arabidopsis syd* mutant plants exhibit reduced *WUS* transcript levels accompanied by abnormal SAM development (Kwon et al., 2005).

The CHR12 and CHR23 ATPases have redundant roles in plant development processes including meristem initiation and maintenance. Plants with strong double mutant *chr12chr23* combinations show embryonic lethality, endosperm defects and failure to initiate stem cell populations in both roots and shoots, whereas plants with weak double mutant *chr12chr23* combinations are small viable plants with noticeable defects in RAM and SAM maintenance (Sang et al., 2012).

A study by Wu et al. (2015) demonstrated a role of chromatin in local developmental progressions in the root and shoot. In the SAM, cells with high auxin levels initiate proteolysis of Aux/IAA leading to the dissociation of the co-repressor complex TOPLESS-HISTONE DEACETYLASE 19 (TPL-HDA19). This enables the recruitment of SYD and BRM to direct the acquisition of flower primordium founder cell fate (Wu et al., 2015). In the RAM, a small group of mitotically inactive cells known as the quiescent center (QC) maintains the root stem cells. BRM was found to act in the *PLETHORA* (*PLT*) pathway to maintain the root stem cell niche by altering the expression of *PIN-FORMED* (*PIN*) genes responsible for auxin distribution in the RAM, as illustrated in **Figure 1**. *brm* mutants exhibit faulty root stem cell niche maintenance, reduced meristem activity and retarded root growth (Yang et al., 2015).

To attain continuous plant growth, cells derived from pluripotent stem cells must undergo asymmetric cell division and expansion. This allows for the generation of lateral organs and the maintenance of stem cell populations for additional growth (Jarillo et al., 2009). The role of BRM in ensuring asymmetric cell division and cell fate determination in both plant and animal stem cells was discussed extensively in a recent review (Pillitteri et al., 2016).

Our current knowledge of mechanisms involving chromatin-mediated regulation of plant stem cell initiation and maintenance remains limited to the few *Arabidopsis* SWI/SNF complexes discussed above. However, emerging evidence suggests complex interconnections among many players including key hormone signaling pathways. This underscores the ability of plants to activate or arrest lateral organ founder cell formation in response to both internal developmental signals and external biotic and abiotic stress signals. These attributes may eventually be harnessed or targeted for crop improvement applications.

## Chromatin Remodeling in Cell Differentiation, Organ Initiation and Development

Stem cells in the SAM initiate organ founder cells that later differentiate into aboveground organ-specific tissues such as leaves and stems (Sinha, 1999; Laux, 2003). Plant roots are formed from a reservoir of undifferentiated cells in the RAM called the root stem cells. Mature plant organs usually maintain relatively undifferentiated cells as a fallback for hormonal stimuli or mechanical injury, helping to generate new tissues and organs through cellular reprogramming (Ikeuchi et al., 2015). Cellular differentiation results from global changes in gene expression patterns and these changes involve many transcription regulators and are epigenetically mediated by chromatin remodeling (Bruex et al., 2012; Taylor-Teeples et al., 2015).

**FIGURE 2:**
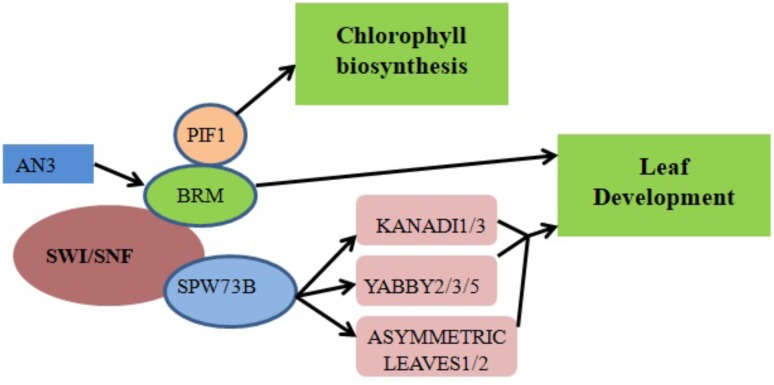
Chromatin remodelers in cell differentiation and organ development regulation. The recruitment of BRM by the transcriptional coactivator AN3 promotes the transcription of genes responsible for leaf development. Direct binding of SWP73B to the promoter regions of *KANADI1/3*, *YABBY2/3/5* and *ASYMMETRIC LEAVES1/2* also controls leaf development. Physical interaction between PIF1 and BRM regulates chlorophyll biosynthesis by affecting the expression levels of associated enzymes.

ANGUSTIFOLIA3 (AN3)/GRF-INTERACTING FACTOR1 (GIF1), a member of the GIF family of transcriptional coactivators, plays a key role in *Arabidopsis* shoot development (Kim and Kende, 2004), cotyledon identity establishment during embryogenesis (Kanei et al., 2012) and leaf size increase resulting from increased cell number (Horiguchi et al., 2005; Lee et al., 2009). Transcriptional coactivators often work together with DNA-binding transcription factors to promote transcription either by recruiting chromatin remodelers or stimulating general complex formation around RNA polymerase II (Pol II). Genetic interaction between AN3 and the *Arabidopsis* SWI/SNF chromatin remodeling complex BRM (**Figure 2**) suggests that AN3 recruits SWI/SNF complexes to promote cell division during leaf development (Vercruyssen et al., 2014). BRM is recruited to its target gene loci via association with the plant-specific H3K27 demethylase RELATIVE OF EARLY FLOWERING 6 (REF6). Through its zinc-finger (ZnF) domains, REF6 recognizes genomic loci harboring a CTCTGYTY motif (Li et al., 2016). A forward genetic screen in *Arabidopsis* indicated that BRM and *SWINGER* (*SWN*) (a key component of Polycomb Group Repressive Complex 2 in plants) antagonistically control vegetative phase change through the temporal expression of miR156 at the nucleosome level. Specifically, the accelerated vegetative phase change of *brm* mutants was accompanied by reduced miR156 expression and increased levels of H3K27me3 at the *MIR156A* locus (Xu et al., 2016).

SWP73 subunits of *Arabidopsis* SWI/SNF CRCs function in various plant development pathways including the regulation of leaf and flower development (Vercruyssen et al., 2014; Sacharowski et al., 2015). For example, SWP73B directly binds to the promoters of the *KANADI1/3*, *YABBY2/3/5* and *ASYMMETRIC LEAVES1/2* genes involved in leaf development (**Figure 2**) (Sacharowski et al., 2015). RNA interference (RNAi) silencing of *SWP73B* leads to dwarfism in *Arabidopsis*, evidencing its role in plant development (Crane and Gelvin, 2007).

A study of the chromatin remodeler protein ZmCHB101, which is the core subunit of maize SWI3, revealed its key roles in normal maize growth and development. ZmCHB101 controls the transcriptional reprogramming of a set of genes involved in gene expression regulation, photosynthesis, metabolic regulation and stress response. The RNAi maize lines generated in the study exhibited improper tassel and cob development and abaxially curling leaves caused by increased bulliform cell numbers (Yu et al., 2016).

Chlorophyll biosynthesis is a critical mark of the transition from heterotrophic to autotrophic growth in plants. Physical interaction between BRM and the transcription factor PHYTOCHROME-INTERACTING FACTOR 1 (PIF1) was shown to regulate chlorophyll biosynthesis in *Arabidopsis* (**Figure 2**). When exposed to light, dark-grown *brm* plants exhibit higher greening rates, reduced protochlorophyllide accumulation and lower levels of reactive oxygen species (ROS) compared to wild-type plants; there is also increased expression of *NADPH:protochlorophyllide oxidoreductase A* (*PORA*), *PORB* and *PORC*, enzymes which accelerate a key step in chlorophyll biosynthesis (Zhang et al., 2017).

The importance of chromatin remodelers for proper plant growth progression and development is exemplified by their involvement in asymmetric cell differentiation and in initiating organ founder cells. Proper plant growth also involves precise developmental phase transitions, and the antagonism between SWI/SNF ATPases and PcG repressive complex components is crucial for these shifts.

## Chromatin Remodeler-Mediated Flowering and Flower Morphogenesis

The precise regulation of the transition from vegetative growth to flowering is paramount for plant reproductive success. This process is characterized by the transition of the vegetative SAM into an inflorescence meristem (IM) and the initiation of floral meristems (FMs) (Sablowski, 2007; Kaufmann et al., 2010). While SAM maintenance ensures indeterminate plant growth, the determinate nature of FMs determines reproductive success, seed development and the yield of agricultural crops (Liu et al., 2011). Complex regulatory networks of transcription factors and chromatin remodelers guide flowering time and flower development while integrating both internal and external signals (Wils and Kaufmann, 2017). These networks comprise photoperiod, vernalization and thermo-sensory pathways for sensing long days, cold winter and ambient temperature, respectively, together with pathways responsive to internal factors, such as the age pathway and the gibberellins (GA) signaling pathway (He, 2012).

In *Arabidopsis*, a repressor complex that consists of the two MADS box transcription factors *MADS AFFECTING FLOWERING 4/5* (*MAF4/5*), *FLOWERING LOCUS C* (*FLC*) and *SHORT VEGETATIVE PHASE* (*SVP*) serves as a negative regulator of flowering time. The components directly repress the expression of the floral pathway integrators *FLOWERING LOCUS T* (*FT*) and *SUPPRESSOR OF OVEREXPRESSION OF CONSTANS 1* (*SOC1*) (Lee et al., 2007; Li et al., 2008; He, 2012). *FRIGIDA* (*FRI*) promotes higher *FLC* levels that inhibit flowering (Michaels and Amasino, 2001) through the recruitment of multiple active chromatin modifications at the *FLC* locus (Choi et al., 2011). Vernalization overrides the *FRI-*mediated activation of *FLC* expression and thereby enables flowering (Kim et al., 2009; Crevilleńn and Dean, 2011).

**FIGURE 3:**
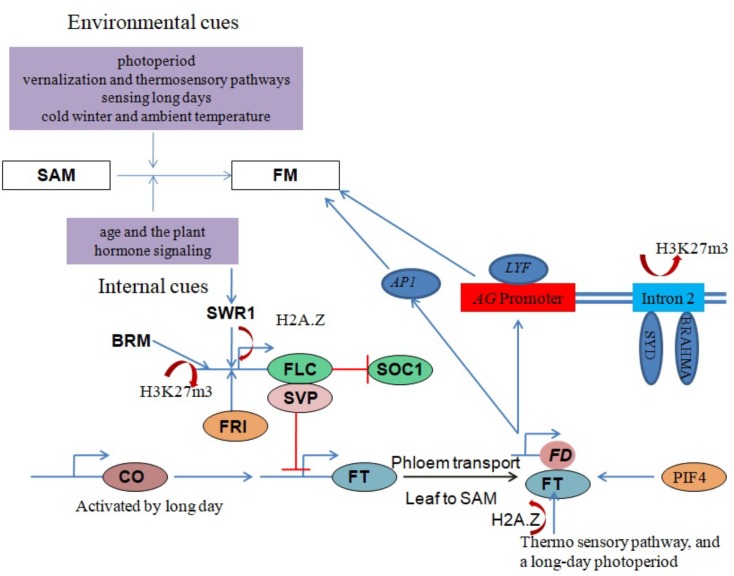
Chromatin modulation in flowering regulation. Red lines with perpendicular bars denote repression, blue arrows indicate activation, and colored ovals represent proteins. Floral transition in the SAM involves IM and FM specification and is regulated by a balance between environmental and internal factors. SYD, BRM and SWR1 chromatin remodeling complexes coordinate with other factors to influence floral development by regulating the expression of critical genes, such as *AG*, *FT*, *FLC* and *SVP*, in response to both external and internal stimuli.

The core subunit components of the *Arabidopsis* SWR1 chromatin remodeling complex (including *PHOTOPERIOD-INDEPENDENT EARLY FLOWERING 1* (*PIE1*), *ACTIN RELATED PROTEIN 6* (*ARP6*) and *SWR1 COMPLEX 6* (*SWC6*)/*SERRATED LEAVES AND EARLY FLOWERING* (*SEF*)) have been shown to play important roles in regulating the proper growth and development of most plant organs. Importantly, SWR1 controls plant development by generating a balance between microRNAs and target mRNAs at the transcriptional level (Choi et al., 2016). It was recently demonstrated that SWR1 regulates gene expression by establishing lowly accessible and highly accessible nucleosome structure at the first nucleosome upstream and downstream of the transcription start site (TSS), respectively (Dai et al., 2017). SWR1 subunit loss-of-function mutants exhibit pleiotropic phenotypes including early flowering, serrated leaves, frequent absence of inflorescence internodes, bushy growth and flowers with altered organ number and size (Choi et al., 2005; Deal et al., 2005; Martin-Trillo et al., 2006; March-Diaz et al., 2007; Qin et al., 2014). H2A.Z deposition around the *FLC* TSS by SWR1 is necessary for *FRI*-meditated activation of *FLC* (**Figure 3**). One line of evidence is that functional disruption of SWR1 prevents H2A.Z deposition at *FLC* chromatin, suppressing its expression and leading to early flowering (Choi et al., 2007; Deal et al., 2007). Additionally, *PIE1* mutations were earlier shown to substantially reduce *FLC* transcript levels, with a concomitant conversion of the winter-annual habit to the rapid flowering summer-annual habit (Noh and Amasino, 2003). A recent study revealed an *Arabidopsis* homolog of the yeast SANT domain protein Swc4/Eaf2 as a novel SWR1-C subunit. SWC4 is a DNA-binding protein that contributes to the recruitment SWR1-C through specific recognition of AT-rich DNA segments in chromatin regions of target genes to deposit H2A.Z. Further, knock-out and knockdown studies showed that *SWC4* is essential for both embryo viability and the control of post-embryonic processes, including flowering time, by repressing transcription of a number of genes including the floral integrator *FT* and key transcription factors (Gomez-Zambrano et al., 2018).

The *At*INO80 complex is required for somatic homologous recombination (HR) and expression regulation of *FLC* and *MAF4/5* by facilitating the enrichment of H2A.Z at their ends. Independent *Atino80-5* and *Atino80-6* mutant alleles display similar pleiotropic phenotypes of small plant size, reduced organ size and late flowering. These observations provide a link between plant responses to environmental and developmental signals and epigenetic mechanisms involved in plant chromatin stability (Zhang et al., 2015).

The SWP73B (BAF60) subunit of the *Arabidopsis* SWI/SNF complex has also been implicated in flowering time control through its involvement in chromatin loop formation at the *FLC* locus (Jegu et al., 2014). SWP73A has been shown to be confined to flowering time modulation under short day conditions, with functional overlap between SWP73A and SWP73B during embryogenesis (Sacharowski et al., 2015).

*SVP*, another key flowering repressor, is highly expressed during the vegetative phase to promote growth (Hartmann et al., 2000) but is down-regulated during the floral transition by the autonomous and GA signaling pathways (Li et al., 2008). *FT* and *SOC1* are subsequently activated to promote flowering (**Figure 3**). One study proposed that BRM controls flowering time in *Arabidopsis* by directly activating *SVP* expression (Li et al., 2015). Genome-wide analysis of H3K27me3, a histone mark associated with gene repression, in *brm* mutant seedlings revealed increased H3K27me3 deposition at several genes including *SVP*, indicating an antagonism between BRM and PcG repressor proteins (Li et al., 2015).

Another key flowering time regulator in *Arabidopsis* activated by *CONSTANS* (*CO*) under long days to induce flowering is the *FT*-encoded florigen protein (FT) (Corbesier et al., 2007). FT moves from the phloem to the SAM and forms a complex with the bZIP transcription factor FD to activate the expression of the FM identity genes *LEAFY* (*LFY*) and *APETALA1* (*AP1*). This activation leads to the formation of the floral primordium, which sequentially generates three types of lateral floral organs (sepals, petals and stamens) (Zik and Irish, 2003; Abe et al., 2005; Wigge et al., 2005). *LFY* recruits SYD and BRM ATPases to induce the expression of the floral homeotic regulator *AGAMOUS* (*AG*) by removing H3K27me3 marks on its second intron (**Figure 3**) (Wu et al., 2012). The SWR1 complex is also involved in *FT* expression regulation, with functional disruption of SWR1 leading to temperature-insensitive *FT* activation and early flowering. An ambient temperature increase from 17 to 27°C causes eviction of the H2A.Z nucleosomes, which enables *FT* transcription by Pol II (Kumar and Wigge, 2010).

Studies of the CHD3 chromatin remodeler *PICKLE* (*PKL*) have highlighted its role in plant reproductive development by promoting crosstalk between the sporophytic and gametophytic generations. Loss of *PKL* in the maternal sporophyte leads to improper development of the *Arabidopsis* female gametophyte, integument and pollen tube, accompanied by delayed ovule and embryo development (Carter et al., 2016).

It was recently shown that during megasporogenesis, somatic cells cooperatively use SWR1 to restrict female reproductive founder cell specification to a single cell in the ovule primordia by incorporating H2A.Z at a particular *WRKY28* nucleosome and promoting expression of the gene (Zhao et al., 2018). The WRKY28 transcription factor is exclusively expressed in hypodermal somatic cells surrounding the megasporocyte and represses those cells from differentiating into functional megasporocytes (Zhao et al., 2018). Other separate findings demonstrated that both BRM and SWR1 are involved in determining inflorescence architecture in response to developmental cues (Zhao et al., 2015; Cai et al., 2017).

The above findings reveal mechanisms involving the recruitment of SWI/SNF ATPases to specific flower development regulatory pathways at an appropriate time. They also emphasize the centrality of SWI/SNF complexes in shaping plant growth and ensuring the continuity of plant viability through generations by precise spatiotemporal control of the relevant developmental processes. Nevertheless, these conclusions about important regulatory mechanisms are generally based on findings from model plants such as *Arabidopsis*. Thus, the understanding of such regulatory mechanisms in agriculturally important crop species remains inadequate and will require further investigation.

## Chromatin Remodelers in Phytohormone Signaling Pathways

Plant hormones are critical for the proper regulation of plant growth and development processes including seed germination, vegetative and reproductive growth and abiotic stress response. The biosynthesis and degradation of plant hormones are also tightly controlled through the transcriptional regulation of target genes. SWI/SNF ATPases are involved in plant hormone responses through physical interaction with hormone signaling pathway components and transcriptional regulators of genes involved in hormone biosynthesis and perception (Sarnowska et al., 2016).

**FIGURE 4:**
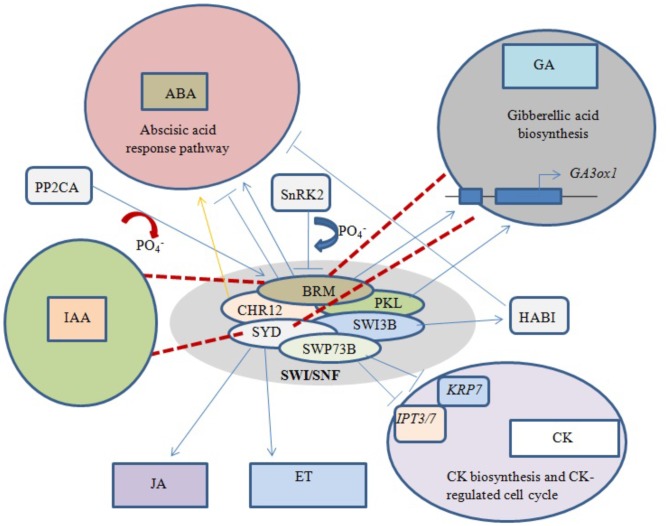
Phytohormone signaling response under chromatin remodeling. Blue arrows represent activation/interaction, blue lines with perpendicular bars denote inhibition, red dashed lines indicate the mutated state of an ATPase, and the yellow arrow represents overexpression. Both BRM and PKL positively regulate GA biosynthesis by activating underlying genes. Phosphorylation of BRM and overexpression of CHR12 both activate the ABA response pathway, while dephosphorylation of BRM and interaction of SWI3B with HABI repress the ABA response. SWP73B negatively regulates CK biosynthesis and CK-mediated cell cycle changes. Mutations in BRM and SYD inhibit GA and IAA biosynthesis, while normal SYD positively regulates JA- and ET-dependent gene expression.

Abscisic acid (ABA) is an important phytohormone that promotes seed dormancy and arrests growth in post-germination embryos under water stress conditions. In the absence of stress stimuli, BRM was initially implicated in repressing the activity of the ABA pathway, with adult *brm* mutants exhibiting increased drought tolerance (Han et al., 2012). It was later revealed that important ABA signaling pathway components physically interact with BRM, leading to post-translational phosphorylation/dephosphorylation of BRM. For example, phosphorylation of BRM by SnRK2 kinases leads to its inhibition, while PP2CA-mediated dephosphorylation restores the ability of BRM to repress the ABA response (**Figure 4**). Moreover, the phosphomimetic BRM mutant shows hypersensitivity to ABA (Peirats-Llobet et al., 2016).

BRM has also been shown to play a direct role in the positive regulation of GA biosynthesis by binding to chromatin near the *GA3ox*1 promoter, thereby activating the gene (**Figure 4**). *Arabidopsis brm* null mutants exhibit a significant decrease in active GA levels (Archacki et al., 2013). Furthermore, transcriptional profiling revealed that most genes involved in the GA and auxin signaling pathways are affected in both *syd* and *brm* null mutants (Bezhani et al., 2007). Previous studies showed that plants over-expressing CHR12 had enhanced growth arrest when exposed to drought or heat stress (Mlynarova et al., 2007). In addition, SYD was linked to ethylene (ET)- and jasmonic acid (JA)-dependent gene regulation (Walley et al., 2008), and the SWI3B subunit was found to interact with a negative regulator of ABA signaling, the PP2C-type phosphatase HAB1 (Saez et al., 2008).

During development, plants transition from the embryonic stage to the seedling stage. Previous studies have characterized the joint effects of PKL and GA in repressing embryonic traits during plant development transitions (Zhang et al., 2008; Zhang et al., 2012). Specifically, *PKL* expression represses the embryonic state by controlling a significant number of GA-responsive genes. *pkl* seedlings exhibit a semi-dwarf phenotype similar to that of GA-response mutants and are able to express embryonic traits under GA-biosynthesis inhibition. In contrast, treatment of *pkl* mutants with GA greatly decreases their characteristic pickle-root phenotype (Henderson et al., 2004). Both PKL and BRM CRCs have been proposed to act as positive regulators of the GA pathway possibly through distinct mechanisms. Unlike *brm* mutants, *pkl* mutant plants have an increased abundance of active GA (Archacki et al., 2013). Recently, PKL was found to positively regulate most GA-mediated developmental processes including promoting vegetative growth (hypocotyl, leaf, and inflorescence stem elongation) and phase transitions (i.e., the juvenile-to-adult and vegetative-to-reproductive transitions) (Park et al., 2017).

Excess cytokinin (CK) production inhibits primary root elongation and weakens lateral root development (Kuderova et al., 2008). SWP73B is one of the accessory subunits of *Arabidopsi*s SWI/SNF complexes shown to positively regulate root development and cell cycle progression in the root meristem by suppressing CK biosynthesis (**Figure 4**). SWP73B negatively regulates the CK biosynthesis genes *ADENOSINE PHOSPHATE-ISOPENTENYLTRANSFERASE* (*IPT3*) and (*IPT7*) and the CK-regulated cell cycle inhibitor *KIP-RELATED PROTEIN7* (*KRP7*) by hindering the deposition of active histone marks on their gene bodies (Jegu et al., 2015).

In aggregate, these findings indicate that both phytohormone biosynthesis and degradation in response to extracellular and intracellular cues partly depend on SWI/SNF chromatin remodeling ATPases. The findings also suggest the role of SWI/SNF ATPases as a hub in plant perception and response to phytohormone signaling as well as hormone crosstalk through direct physical interactions with hormone signaling pathway components. The outcomes of these interactions influence the plant response to abiotic stress, enabling plant adaptability under changing climates.

## Concluding Remarks and Future Perspectives

Chromatin remodeling has been extensively studied in the context of various regulatory and developmental processes in a number of eukaryotic organisms, including humans, mice, yeast, fruit fly and *Arabidopsis*. Phylogenetic analysis suggests that the chromatin remodeling machinery and their modes of operation are evolutionarily conserved across eukaryotes. However, SWI/SNF subunit loss-of-function studies have uncovered varied organism-specific phenotypes. These phenotypes also depend on the strength of a given mutation, leading to phenotypic differences among single and double mutants of SWI/SNF subunit genes. The functional redundancy of some SWI/SNF complex subunits also necessitates further in-depth analyses of their interaction patterns in different plant development pathways.

As discussed above, a number of plant regulatory and developmental transitions are controlled by chromatin modifiers through complex pathways in response to both endogenous and environmental factors. These precisely orchestrated mechanisms are heritable, underscoring their importance for plant survival in changing habitats. Moreover, the dynamics of these epigenetic controls go hand in hand with changes in plant growth conditions. Positive attributes that could potentially be integrated into plant breeding schemes are of interest to many plant scientists and breeders insofar as they may yield varieties that can withstand adverse weather conditions, early flowering or various biotic stresses and maximize nutrient utilization for improved yield. High-throughput technologies together with the vast genomic data presently available in public repositories should facilitate comparative and functional studies aimed at plant improvement. Future studies in other species of interest (e.g., rice and other crops) will likely build upon the findings on ATP-dependent CRCs in *Arabidopsis* and more recently in maize. Plant-specific molecular and genetic approaches should also characterize regulatory differences between developmental stages and under different growth conditions for a more comprehensive understanding of the involvement of SWI/SNF chromatin modifiers in plant growth and development.

## General Outlook

The following research questions and topics are of particular interest in terms of how CRCs may be involved in various processes either directly or indirectly related to plant development.

(1) Involvement of SWI/SNF ATPases in flowering regulation in the temperate legume model plant *Medicago truncatula*, whose flowering is prompted by winter cold and long-day photoperiods despite the absence of an *FLC* gene.
(2) The role played by chromatin remodelers in oxidative stress remediation processes resulting from various metabolic activities in plant cells other than DNA double-strand break repair.
(3) Besides their known function in the phosphate starvation response, there is a need to ascertain other roles of SWI/SNF remodelers in mediating the uptake of essential micro- and macronutrients from the soil in conjunction with nutrient transporter gene families and rhizosphere microbial activities.
(4) The mechanism by which the chromatin remodeling complex SWR1 mediates chromatin structural dynamics by replacing the core H2A-H2B histone dimer with the H2A.Z-H2B dimer in octamers is well characterized. However, the molecular mechanisms underlying the reverse process remain elusive.
(5) The specific roles of accessory subunits of *Arabidopsis* SWI/SNF CRCs in regulating plant development are only beginning to be understood. The latest example is SWP73 involvement in the cell cycle, leaf development, flowering time and embryogenesis. Much remains to be uncovered in this regard.

## Author Contributions

SO and SC conceived the idea, wrote the manuscript, and drafted the figures. SP designed and organized figures and revised the manuscript. WL, MY, MA, and HZ revised the manuscript and organized the figures. YQ conceived the idea and revised and organized the manuscript.

## Conflict of Interest Statement

The authors declare that the research was conducted in the absence of any commercial or financial relationships that could be construed as a potential conflict of interest.
